# Pacemaker Lead Displacement: Mechanisms And Management

**Published:** 2003-10-01

**Authors:** Beatriz Fuertes, Jorge Toquero, Ramon Arroyo-Espliguero, Ignacio F Lozano

**Affiliations:** Department of Cardiology, Hospital Puerta de Hierro, Madrid. Spain

## Introduction

Pacemaker lead displacements can be defined as any other pacemaker position change, whether the functionality of the pacemaker is affected or not. However, only those displacements that provoke a malfunction in the pacing system are clinically relevant. Chronologically speaking, there are early displacements, which occur within the first six weeks after implantation, and late displacements, after this period of time [1]. Early displacements are more frequent than late displacements and they usually affect atrial leads. The incidence of early displacements is 1% in VVI pacemakers and 5.2% in DDD pacemakers (3.8% of the cases affecting atrial leads and 1.4% ventricular leads). Acceptable displacement rates should probably be less than 1 percent for ventricular leads and no more than 2 to 3 percent for atrial leads. These values are higher in biventricular pacing devices, related to coronary sinus lead displacement. Early lead displacements are the most frequent cause of reintervention, involving atrial leads in the majority of cases. After the first six weeks, late displacements are remarkable and they can rarely be related to a specific event [2].

Long-term stability in epicardial systems, though it may seem different, is similar to that of endocardial systems [3]. The difference in the number of complications is not significant between both systems. Epicardial leads are implanted using median sternotomy, and sutured to the epicardial surface of the heart. Pacemaker generator is located in the abdominal cavity and the lead tunnelized through the anterior aspect of the diaphragm. Globally speaking, complications rate is estimated among 5% to 13%.

Among the complications that may arise after the implantation of a permanent endocardial or epicardial cardiac pacing device, or a cardioverter defibrillator, lead displacements, together with lead fractures or retractions, usually take place within the first months after implantation. On the other hand, faults in the isolating material, in the adapter or in the connectors may arise later.

Dislodgement has been classified as “macrodislodgement” and “micro-dislodgement”. Macrodislodgement is radiographically evident, microdislodgement is not, and they constitute a particular case. They are considered as minimal displacements in the lead tip, not evident in chest radiographs, but enough to produce an increase in capture threshold and eventually a loss of capture while keeping normal lead impedance values or changing the initial values minimally. This is due to the fact that current density, responsible for the myocardial depolarization provoked by the pacemaker, is directly proportional to the current intensity (amperage) and inversely proportional to the distribution area. Since the area size increases proportionally to the radius square, the density will decrease proportionally to that square, this one being the distance between the current distribution center and the myocardium to be depolarized, (that is to say, the sum of the lead radius and the thickness of the fibrosis caused by the lead).

## Etiology

Information about the causes of lead displacement is scarce and it is often difficult to relate lead displacements to a specific etiology. Among some of them, we may name the following:

Twiddler’s Syndrome : a rare complication observed both in patients with implanted pacemakers and defibrillators. First described in 1968, refers to permanent malfunction of a pacemaker due to the patient’s manipulation of the pulse generator. The patient, inadvertently or deliberately, turns and rotates the generator on its long axis and, because of traction, causes the lead displacement. Most patients with such a problem are middle-aged obese women whose surgical pocket is larger than the size of the generator together with the presence of loose subcutaneous tissue [4-6]. Ipsilateral phrenic nerve can be stimulated, resulting in diaphragmatic pacing and sensation of abdominal pulsations. Even rhythmic arm twitching has been described [7], related to pacing the brachial plexus.
Reel’s Syndrome : It is similar to the Twiddler’s Syndrome. In this case, the patient rotates the generator on its transverse axis rolling the lead around the generator and provoking a lead displacement. Chest radiography is crucial to diagnose this kind of complication [8].
Direct trauma over the system : this may produce not only a lead or connection fracture but also a system displacement [9], leading to macro or micro-displacements. Intense respiratory therapy is one of the etiologies that may lead to micro-dislodgment, specifically “Clapping” [10,11]. It consists of manual application of rhythmic percussion to the patient’s chest wall to break up and detach the accumulated bronchial wall secretions and requires considerable energy. Other passive techniques, such as vibration or vibrating pressure are recommended instead.

Any significant trauma over the system, because of traction, may produce a micro-displacement resulting in discontinuous capture or even loss of capture with the programmed output voltage. When checking the device, sensing, lead impedance and battery status are usually correct. Leads appear well in place in chest radiographies making difficult the diagnosis of the problem. In some cases, however, the use of fluoroscopy will help us to see that the lead is free in the right ventricle.

## Diagnosis

In general, the first signs of pacemaker’s malfunction will show in hospital. Depending on the pacemaker-dependency of the patient and on the degree of loss of capture, moderate dizziness or lightheadedness episodes and even repeated syncope’s may be reported. Other times, the only symptom reported is that derived from extra cardiac pacing. Patients may experience discomfort in the chest area or have hiccups due to phoenix nerve stimulation, mainly on the right side because of anomalous position of the atrial lead. Hemidiaphragm contraction synchronized with pacemaker pacing may occur, evidenced by the palpation of the upper quadrants of the abdomen or even by mere eye inspection, which can be eliminated or reduced if pacemaker output is programmed to a lower value. Left intercostal pacing is always caused by right ventricle perforation by the lead [12].

There are many etiologies [13] on pacing failures in permanent pacing systems (Table 1) but many of them are out of the scope of this work and we will focus from now on those provoked by lead displacements.

The symptoms referred by the patient will give us the first approach to device malfunction. The most useful and cost-effective tools to detect pacemaker malfunctions related to lead displacement are the ECG and the chest radiography. After that, lead status can be established interrogating the device through the programmer, measuring lead impedance and determining the pacing threshold (Table 2).

**Figure 1:**
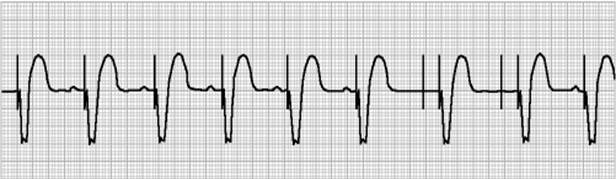
Transient sinus arrest in a dual chamber pacemaker, depicting loss of atrial capture due to micro-dislodgement with normal P wave sensing

**Figure 2:**
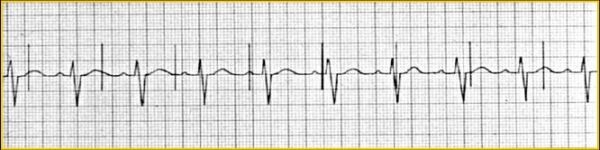
Loss of ventricular sensing and capture due to lead displacement

The ECG constitutes a valuable tool when evaluating a patient with suspected lead displacement. Loss of capture with adequate sensing points to micro-dislodgment (Figure 1), whereas combination of loss of capture and inadequate sensing suggest lead displacement (Figure 2).

**Figure 3:**
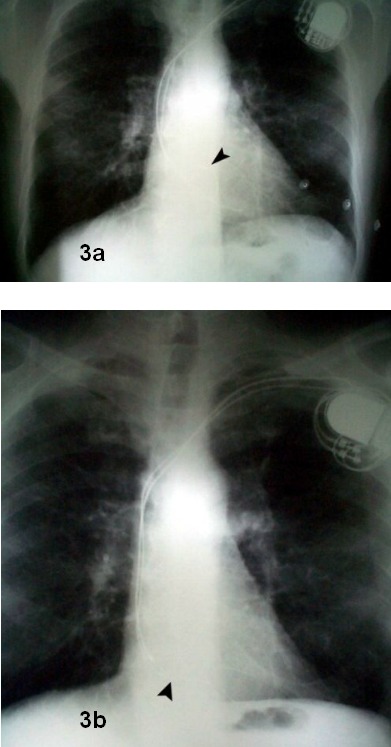
Posteroanterior chest x-ray film. Top (**A**): radiograph obtained 24 hours after pacemaker implantation. Arrowhead shows atrial lead tip inside the right atrial appendage. Bottom (**B**): radiograph obtained three months later, showing displacement of atrial lead towards tricuspid annulus. However, diagnosis is not evident from the posteroanterior view alone, due to superimposition of structures and radiograph densities.

**Figure 4:**
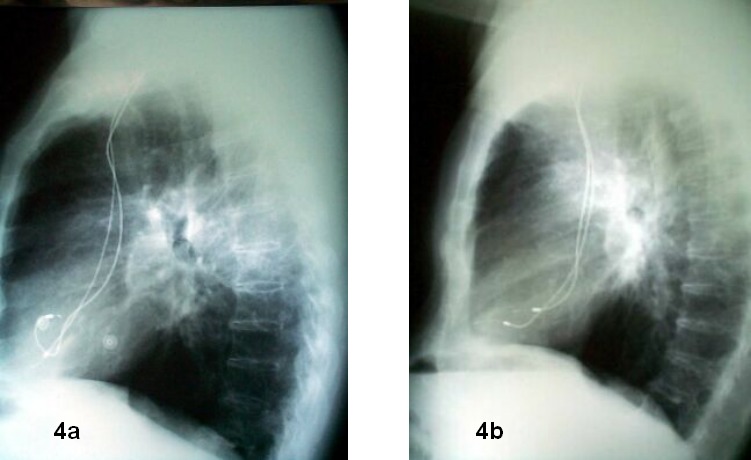
Lateral chest x-ray film (same patient as in figure 3): left (A): 24 hours post implantation. Right (B): 3 months later. Atrial lead displacement diagnosis is now straightforward. The example emphasizes the relevance of two chest views available to correctly evaluate lead displacement.

The ECG allows us to determine permanent or non-permanent capture failures and even the absence of pacing artifact. This finding opens a wide range of likely etiologies that can be limited using other complementary tests. When evaluating the chest X ray, it is crucial to have two views available, lateral and posteroanterior, to determine whether leads have been placed correctly (see Figure 3 and 4). Passive fixation atrial leads are usually placed in the right appendage, and its trabeculation is important to provide more stability. In special circumstances, it may be interesting to perform an atrial implantation far from the right ventricle, either on the free wall or the interatrial septum. This is used either to avoid interferences in the atrial channel, as it happens in far-field potentials in double-chamber defibrillators, or to reduce the incidence of atrial fibrillation. An active fixation lead is used in these situations. Passive fixation ventricular leads are placed in the apical area, known to be trabeculated and therefore, stable for lead implantation. Although ventricular leads have been exceptionally placed on the right ventricle outflow tract, the presence of a lead tip at that position indicates a clear displacement in most occasions. In posteroanterior chest radiographies, a lead should be placed inside the right ventricle oriented to the apex. In the lateral view, the apical placement should be verified with an anterior orientation. In case of frank displacements, the lead can be sometimes seen completely free in the ventricle, although that is not the case in most occasions. Micro-displacements as short as 1-2 mm cannot be seen in the X ray. It is often more difficult to see displacements in left ventricular pacing electrodes since there is no clear cut anatomical structure to define its position. It is then crucial to have a baseline radiography available in the period 24-48 hours after implantation to use as a reference for subsequent evaluations.

 After the clinical evaluation and first complementary tests have lead us to suspect that a displacement has occurred, the next step is to interrogate the device: a displaced lead is characterized by an abnormal reduction in impedance values and a higher capture threshold. In micro-displacements, usually harder to detect, an increase in capture threshold and a normal impedance, or an unchanged impedance with respect to the previous one, can be observed. In some occasions, we may find important variations in capture threshold and pacing impedance values at different stages depending on the degree of myocardial contact of the displaced lead.

## Treatment

The approach to lead displacement is going to be different depending on time from implantation, patient clinical status, pacemaker dependency, lead displaced (atrial or ventricular, active or passive fixation) and degree of malfunction of the device. In early displacements, reopening the pouch and lead reposition are possible since the distal end of the lead has not been caught and fixed by the endocardial fibrous reaction [14], allowing its manipulation.

In late displacements, surgical repositioning is often not feasible. In these cases, a solution is to implant a new lead in the chamber in which displacement has occurred canceling the previous one. Lead repositioning via percutaneous access is a less aggressive option that has provided also good results in cases reported. This technique was initially described to reposition J-shaped passive fixation atrial leads [15]. A quadripolar catheter with detectable curve must be advanced up to the atrial lead through a femoral vein approach. Then, the femoral catheter is removed while performing a clock-wise turn at the same time. This allow us to pull the electrode curve until it is released and goes back to its normal J position contacting with the higher portion of the right atrium. After these proceedings, it must be checked whether operating parameters are normal.

Transvenous proceedings are easy, fast (average 15 min.) and provide good results (evaluated through verified stability after one to six months). Authors conclude that can be successfully carried out using a percutaneous catheter through the femoral vein. They are straightforward, safe and no new surgical intervention is needed, even in patients with dilated right atrium, a factor that may negatively affect lead stability. Adverse effects of this technique are derived from the femoral vein approach. For this reason, percutaneous lead reposition should always be considered in most patients before performing an open surgery intervention.

However, prevention and not treatment is the most important aspect when dealing with lead displacements. Several facts have to be taken into account in order to avoid lead displacements:

- Surgical implantation must be adequate from a technical point of view, creating a small surgical pocket to avoid generator displacements that may put pressure on the lead and displace it. Lead fixation to vein access and generator suturing to the fascia will help to keep the system stable [16].
- Using active fixation leads, particularly in those patients whose chambers could have no proper trabeculation as it is the case in dilated cardiomyopathy or atrial dilatation [17].
- Always verify the position of the leads in the period 24-48h after implantation using chest X ray in posteroanterior and lateral views.
- Consider subpectoral implantation in aged obese patients whose flabby subcutaneous tissue may allow for progressive generator displacement inside the subcutaneous pouch and eventually progressive lead displacement. Our group previously described [18]. the utility of placing the pulse generator beneath the pectoral muscle and securing it to the fascia, to avoid recurrent Twiddler’s Syndrome. Technology development and improvement as well as reduction in generators’ size and weight have contributed to reduce this complication.
- Introduce the generator in a Dacron pouch: although it is not a generally accepted method, it should be taken into account in cases in which pouch erosion, migration or manipulation have been observed.

## Conclusion

In spite of its importance, studies on the incidence and handling of lead displacement are surprisingly scarce. From reported cases related to this problem and its etiologies, we can conclude that, even using state of the art electrode technology and the most adequate surgical technique, lead displacement is a complication that has to be kept in mind for an early approach whenever feasible. Clinical evaluation of the patient will suggest the diagnosis. Together with ECG and chest X ray, a correct diagnosis can be made in the majority of cases. Device interrogation through corresponding programmer is helpful in doubtful situations, as micro-dislodgements. Our best tool to prevent lead displacement is the use of careful and adequate surgical implantation techniques. Percutaneous techniques could also allow us to reposition leads in a less aggressive way maintaining an adequate success rate and reducing the number of complications. When this is not feasible, surgical approach is necessary to manage the problem.

## Figures and Tables

**Table 1:**
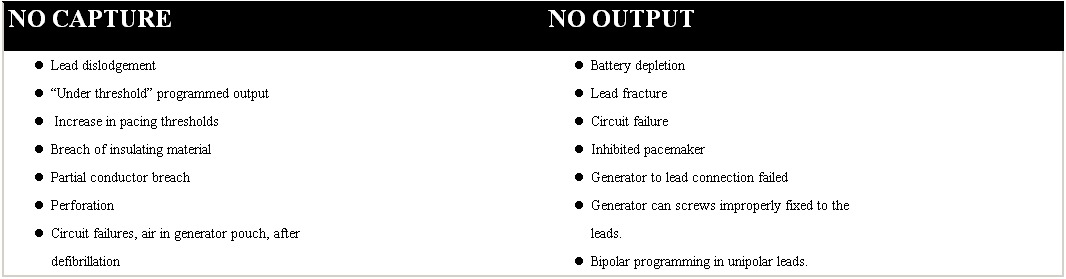
Most frequent causes for pacing failures in permanent cardiac pacing systems

**Table 2:**
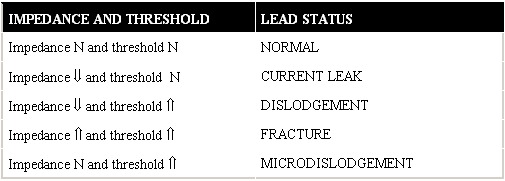
Diagnostic approach to the most frequent lead malfunctions using impedance and threshold values
